# A Facile Synthesis of Deaza-Analogues of the Bisindole Marine Alkaloid Topsentin

**DOI:** 10.3390/molecules18032518

**Published:** 2013-02-26

**Authors:** Anna Carbone, Virginia Spanò, Barbara Parrino, Cristina Ciancimino, Orazio A. Attanasi, Gianfranco Favi

**Affiliations:** 1 Dipartimento di Scienze e Tecnologie Biologiche Chimiche e Farmaceutiche, Università degli Studi di Palermo, Via Archirafi 32, 90123 Palermo, Italy; 2 Dipartimento di Scienze Biomolecolari, Università degli Studi di Urbino “Carlo Bo”, Via I Maggetti 24, 61029 Urbino, Italy

**Keywords:** topsentin, bis-indole alkaloids, antitumor activity, ethyl 1-[(*tert*-butoxycarbonyl)amino]-2-methyl-5-(1-methyl-1*H*-indol-3-yl)-4-[(1-methyl-1*H*-indol-3-yl)-carbonyl]-1*H*-pyrrole-3-carboxylates

## Abstract

A series of substituted ethyl 1-[(*tert*-butoxycarbonyl)amino]-2-methyl-5-(1-methyl-1*H*-indol-3-yl)-4-[(1-methyl-1*H*-indol-3-yl)carbonyl]-1*H*-pyrrole-3-carboxylates were prepared in excellent yields (82-98%) by one-pot reactions between β-dicarbonyl compounds **12a**–**e** and 1,2-diaza-1,3-diene (DD) **13**. Derivatives **10a**,**c**–**e**, deazaanalogues of the bis-indole alkaloid topsentin, screened by the National Cancer Institute (Bethesda, MD, USA) in the *in vitro* one dose primary anticancer assay against a panel of about 60 human tumor cell lines, showed no significant activity, with the exception of compound **9e**, which showed moderate activity against the HOP-92 cell line of the non small cell lung cancer sub-panel and the SNB-75 cell line of the CNS sub-panel.

## 1. Introduction

**Figure 1:**
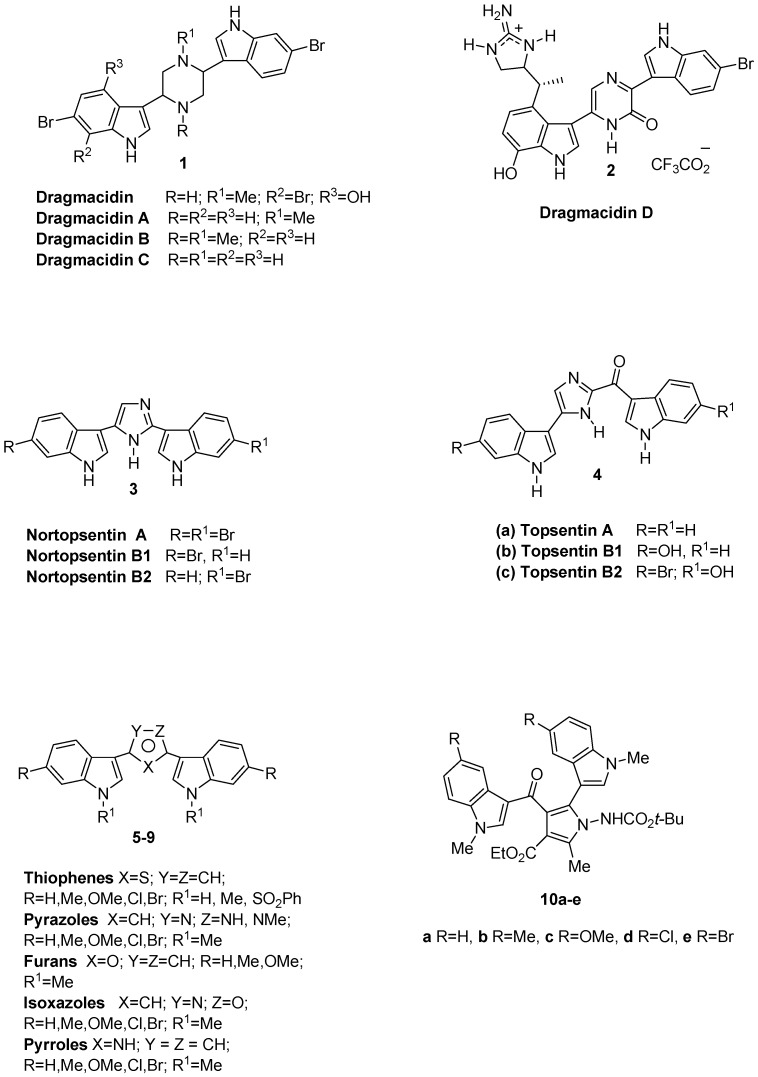
Bis-indolyl alkaloids.

Marine invertebrates represent a very important source of bioactive molecules. In particular, bis-indole alkaloids (Figure 1), characterized by two indole units bound to a spacer through their 3 position, constitute a class of deep-sea sponge metabolites with potent antiinflammatory, antimicrobial, antiviral and antitumor biological activity [1,2,3,4]. The first isolated four dragmacidins **1**, containing the six membered piperazine heterocyclic ring, were isolated from a large number of deep water sponges including *Dragmacidon*, *Halicortex*, *Spongosorites*, *Hexadella* and the tunicate *Didemnum candidum* and showed, among other biological properties, modest cytotoxic activity [5,6,7]. Successively, more complex components of this family such as dragmacidin D (**2**), having a pyrazinone moiety as a spacer, exhibited varied biological properties such as inhibition of serine-threonine protein phosphatases, antiviral, antimicrobial and anticancer activities [8,9]. Nortopsentins A–C **3**, with imidazole as a five membered ring spacer, were isolated from the marine sponge *Spongosorites ruetzleri* and showed *in vitro* cytotoxicity against P388 cells and antifungal activity against *Candida albicans* [10,11]. Topsentins **4** [12], having a bis(indolyl)imidazole skeleton (Figure 1), exhibited antiviral and antitumor activity. In particular compounds **4a** and **4b** had *in vitro* activity against HSV-1, vesicular stomatitis virus (VSV), and corona virus A-59 [13]. Topsentin **4a** showed *in vitro* cytotoxicity against P388 murine tumor cells (IC_50_ 3.0 µg/mL) and human tumor cells (HCT-8, A-549, T47D 20 µg/mL) and *in vivo* activity against P388 (T/C 137%, 150 mg/kg) and B16 melanoma (T/C 144%, 37.5 mg/kg) [14]. Compounds **4b** and **4c** exhibited antiproliferative activity against human broncopulmonary cancer cells (NSCLC-N6) with IC_50_ values of 12.0 and 6.3 µg/mL, respectively [2]. Due to these interesting biological activities, there has been a rapid growth of interest in the synthesis of this class of compounds and their analogues [15,16,17,18,19].

We have recently reported the synthesis and the antitumor activity of bis-indolyl thiophene [20] **5**, pyrazoles [21] **6**, furans [22] **7** and isoxazoles [22] **8** (Figure 1) that showed inhibitory activity against a wide range of human tumor cell lines, generally in the micro- and submicromolar range. Even more recently bis-indolyl pyrroles [23] **9** have exhibited concentration-dependent antitumor activity towards a panel of 42 human tumor cell lines, with mean IC_50_ values of 1.54 µM and 0.67 µM, respectively. Moreover, investigating human tumor xenografts in an *ex vivo* clonogenic assay revealed selective antitumor activity.

Thus, in our effort to search for novel antitumor compounds, and with the aim to develop more potent and selective agents and considering the good antiproliferative activity of bis-indolyl pyrroles, we designed further new analogues of the indole alkaloid topsentin. We report herein a synthesis of ethyl 1-[(*tert*-butoxycarbonyl)amino]-2-methyl-5-(1-methyl-1*H*-indol-3-yl)-4-[(1-methyl-1*H*-indol-3-yl)carbonyl]-1*H*-pyrrole-3-carboxylates derivatives **10a**–**e** (Figure 1), in which the imidazole unit moiety of topsentin was replaced by a highly functionalized pyrrole ring and the two indolyl portions are placed in adjacent positions.

## 2. Results and Discussion

**Scheme 1:**
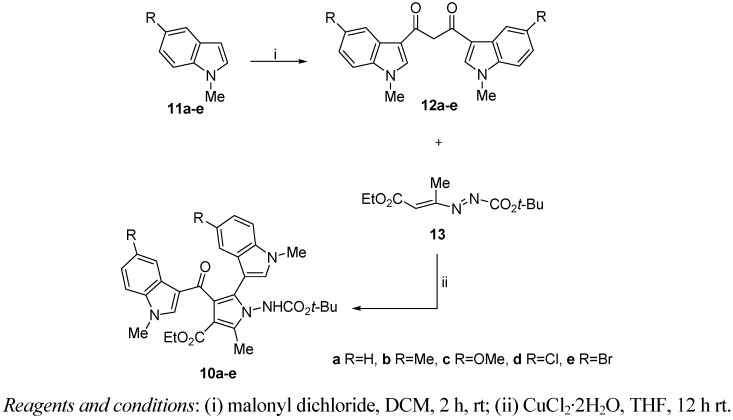
Synthesis of derivatives **10a**–**e**.

The key intermediates for the synthesis of ethyl 1-[(*tert*-butoxycarbonyl)amino]-2-methyl-5-(1-methyl-1*H*-indol-3-yl)-4-[(1-methyl-1*H*-indol-3-yl)carbonyl]-1*H*-pyrrole-3-carboxylates **10a**–**e** are the β-diketones **12a**–**e** prepared in good yields (45–70%) from *N*-methylindoles **11a**–**e** by a Friedel-Crafts reaction using malonyl dichloride in dichloromethane, as previously reported [22] (Scheme 1).

One-pot reaction of the latter with 1,2-diaza-1,3-diene (DD) **13** in tetrahydrofuran and in the presence of CuCl_2_∙2H_2_O as catalyst furnished the corresponding ethyl 1-[(*tert*-butoxycarbonyl)amino]-2-methyl-5-(1-methyl-1*H*-indol-3-yl)-4-[(1-methyl-1*H*-indol-3-yl)carbonyl]-1*H*-pyrrole-3-carboxyl-ates **10a**–**e** in excellent yields (82–98%).

**Scheme 2:**
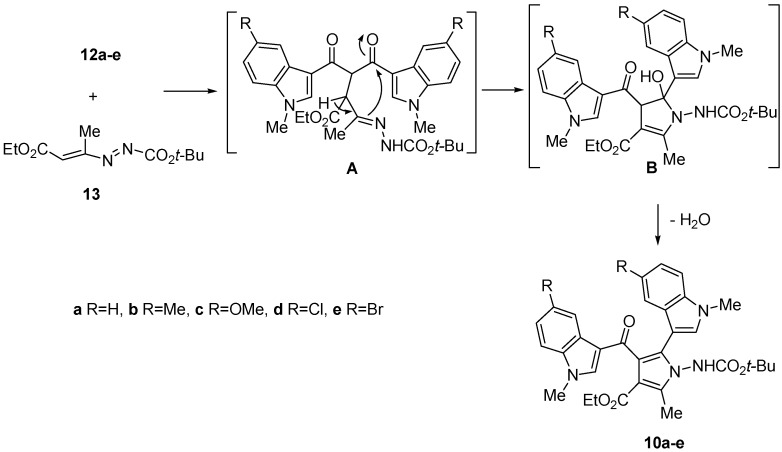
Mechanism.

The mechanism of the latter reaction involves the copper-promoted attack of the active carbon atom of diketones **12a**–**e** at the terminal carbon of the heterodiene system of DD (**13**) to give an adduct intermediate **A**, followed by an intramolecular cyclization reaction to generate the ethyl 1-[(*tert*-butoxycarbonyl)amino]-5-hydroxy-2-methyl-5-(1-methyl-1*H*-indol-3-yl)-4-[(1-methyl-1*H*-indol-3-yl)carbonyl]-4,5-dihydro-1*H*-pyrrole-3-carboxylate derivative **B**. Then, the spontaneous loss of a water molecule provides the related aromatic pyrrole (Scheme 2). This five membered ring closure is substantiated by several papers previous presented by some of us [24,25].

Four pyrrole derivatives **10a**,**c**–**e** were selected for a single dose concentration, *in vitro* disease-oriented antitumor screenings against the full NCI panel of about 60 human tumor cell lines that have grouped in disease sub-panels including leukaemia, non-small lung, colon, central nervous system, melanoma, ovarian, renal, prostate, and breast tumors cell lines [26]. The results obtained take into consideration the percent growth of the treated cells. The results revealed that compound **10e**, tested at the concentration of 10^−4^ M, showed a growth of −27.31% against the SNB-75 cell line of the CNS cancer sub-panel and 4.73% against the HOP-92 of the non-small cell lung cancer sub-panel. The other tested compounds showed no significant activity.

## 3. Experimental

### 3.1. Chemistry

#### 3.1.1 General Procedures

All the commercially available reagents and solvents were used without further purification. 1,2-Diaza-1,3-diene (DD, **13**) was synthesized as a mixture of *E/Z* isomers as previously reported [27,28,29]. Column chromatography was performed with Merck silica gel 230–400 mesh ASTM or with a Büchi Sepacor chromatography module (a prepacked cartridge system). TLC analysis was performed on pre-loaded (0.25 mm) glass-supported silica gel plates (Kieselgel 60); compounds were visualized by exposure to UV light and by dipping the plates in 1% Ce(SO_4_)·4H_2_O, 2.5% (NH_4_)_6_Mo_7_O_24_·4H_2_O in 10% sulfuric acid followed by heating on a hot plate. ^1^H-NMR and ^13^C-NMR spectra were recorded in DMSO-*d_6_* or CDCl_3_ solution on 200 (Bruker AC) or 400 (Varian Mercury) MHz instruments. Proton and carbon spectra were referenced internally to solvent signals, using values of δ = 2.49 ppm for proton (middle peak) and δ = 39.50 ppm for carbon (middle peak) in DMSO-*d*_6_ and δ = 7.26 ppm for proton and δ = 77.00 ppm for carbon (middle peak) in CDCl_3_. The following abbreviations are used to describe peak patterns where appropriate: s = singlet, d = doublet, t = triplet q = quartet and m = multiplet. All coupling constants (*J*) are given in Hz. All melting points were taken on a Büchi-Tottoli capillary apparatus and are uncorrected; IR spectra were determined in bromoform or nujol with a Jasco FT/IR 5300 spectrophotometer. Mass spectra were recorded in the EI mode (70eV) on a Shimadzu GC-MS QP5050A spectrometer. Elemental analyses (C, H, N) were within ±0.4% of the theoretical values.

##### 3.1.1.1. General Procedure for the Preparation of 1,3-bis(indol-3-yl)propane-1,3-diones **12a**–**e**

A solution of malonyl dichloride (1 mL, 10 mmol) in DCM (10 mL) was added dropwise to a cold solution of the appropriate *N*-methylindole **11a**–**e** (20 mmol) in DCM (20 mL). The reaction mixture was stirred for 2 h at room temperature. The mixture was then added to 5% aqueous sodium carbonate and shaken for 2 min. This was extracted with DCM, dried and evaporated. The residue was purified by chromatography eluting with DCM/Ethyl acetate 9/1 to give derivatives **12a**–**e**. Analytical and spectroscopic data are reported elsewhere [12].

##### 3.1.1.2 General Procedure for the Preparation of Ethyl 1-[(tert-butoxycarbonyl)amino]-2-methyl-5-(1-methyl-1H-indol-3-yl)-4-[(1-methyl-1H-indol-3-yl)carbonyl]-1H-pyrrole-3-carboxylates **10a**–**e**

To a solution of the appropriate 1,3-bis(indol-3-yl)propane-1,3-dione **12a**–**e** (1.0 mmol) in THF (5 mL), 1,2-diaza-1,3-diene (DD, **13**) as a mixture of E/Z isomers (1.0 mmol) and CuCl_2_∙2H_2_O (0.5 mmol) were added. The reaction was stirred under magnetic stirring at room temperature for 12 h, until the disappearance of the DD (as monitored by TLC). The crude mixture was concentrated under reduced pressure and the products **10a**–**e** were purified by column chromatography on silica gel (eluent: ethyl acetate/cyclohexane = 1:1).

*Ethyl 1-[(tert-butoxycarbonyl)amino]-2-methyl-5-(1-methyl-1H-indol-3-yl)-4-[(1-methyl-1H-indol-3-yl)carbonyl]-1H-pyrrole-3-carboxylate* (**10a**). Pale yellow solid; yield: 98%; mp: 169–173 °C; IR (nujol) ν 3263, 1765, 1682, 1626 cm^−1^; ^1^H-NMR (400 MHz, DMSO-*d_6_*) δ 0.75 (t, *J* = 7.0 Hz, 3H), 1.34 (s, 9H), 2.39 (s, 3H), 3.68 (s, 3H), 3.69 (s, 3H), 3.87 (q, *J* = 7.0 Hz, 2H), 6.91 (t, *J* = 7.2 Hz, 1H), 7.08 (t, *J* = 7.2 Hz, 1H), 7.12 (t, *J* = 7.2 Hz, 1H), 7.17 (d, *J* = 7.2 Hz, 1H), 7.20 (s, 1H), 7.33 (d, *J* = 8.0 Hz, 1H), 7.39 (d, *J* = 8.0 Hz, 1H), 7.45 (d, *J* = 8.0 Hz, 1H), 7.52 (s, 1H), 8.01 (d, *J* = 8.0 Hz, 1H), 10.20 (s, 1H); ^13^C-NMR (100 MHz, DMSO-*d_6_*) δ 10.4 (q), 13.5 (q), 27.8 (q), 32.4 (q), 32.8 (q), 58.9 (t), 80.7 (s), 102.6 (s), 108.9 (s), 109.7 (d), 110.3 (d), 117.6 (s), 119.2 (s), 119.8 (d), 121.3 (2C, d), 121.6 (d), 122.5 (d), 122.8 (d), 125.9 (s), 127.6 (s), 130.6 (d), 135.8 (s), 136.1 (s), 137.1 (s), 137.6 (d), 154.6 (s), 163.8 (s), 186.5 (s). MS: *m/z* (%) = 554 [M^+^] (48), 498 (100), 454 (78), 392 (71), 367 (28), 338 (28), 158 (92). Anal. Calcd for C_32_H_34_N_4_O_5_: C, 69.30; H, 6.18; N, 10.10. Found: C, 69.47; H, 5.93; N, 10.22.

*Ethyl 1-[(tert-butoxycarbonyl)amino]-2-methyl-5-(1,5-dimethyl-1H-indol-3-yl)-4-[(1,5-dimethyl-1H-indol-3-yl)carbonyl]-1H-pyrrole-3-carboxylate* (**10b**). Pale yellow solid; yield: 94%; mp: 186–190 °C; IR (nujol) ν 3247, 1744, 1684, 1628 cm^−1^; ^1^H-NMR (400 MHz, DMSO-*d_6_*) δ 0.79 (t, *J* = 7.0 Hz, 3H), 1.34 (s, 9H), 2.21 (s, 3H), 2.35 (s, 3H), 2.38 (s, 3H), 3.64 (s, 3H), 3.65 (s, 3H), 3.89 (q, *J* = 7.0 Hz, 2H), 6.88 (d, *J* = 8.0 Hz, 1H), 6.99 (d, *J* = 7.2 Hz, 1H), 7.11 (s, 1H), 7.20 (d, *J* = 8.0 Hz, 1H), 7.23 (s, 1H), 7.26 (d, *J* = 8.0 Hz, 1H), 7.49 (s, 1H), 7.81 (s, 1H), 10.16 (s, 1H); ^13^C NMR (100 MHz, DMSO-*d_6_*) δ 10.4 (q), 13.6 (q), 21.0 (q), 21.1 (q), 27.8 (q), 32.4 (q), 32.7 (q), 58.9 (t), 80.6 (s), 102.1 (s), 108.7 (s), 109.3 (d), 109.9 (d), 117.2 (s), 119.6 (d), 121.1 (d), 122.8 (d), 123,0 (s), 123.9 (d), 125.9 (s), 126.1 (s), 127.6 (s), 128.0 (s), 130.4 (d), 130.6 (s), 134.5 (s), 135.5 (s), 135.7 (s), 137.6 (d), 154.5 (s), 163.8 (s), 186.5 (s). MS: *m/z* (%) = 582 [M^+^] (45), 526 (100), 482 (98), 420 (53), 172 (68). Anal. Calcd for C_34_H_38_N_4_O_5_: C, 70.08; H, 6.57; N, 9.62. Found: C, 69.82; H, 6.75; N, 9.75.

*Ethyl 1-[(tert-butoxycarbonyl)amino]-2-methyl-5-(5-methoxy-1-methyl-1H-indol-3-yl)-4-[(5-methoxy-1-methyl-1H-indol-3-yl)carbonyl]-1H-pyrrole-3-carboxylate* (**10c**). White solid; yield: 83%; mp: 202–206 °C; IR (nujol) ν 3148, 1746, 1731, 1705 cm^−1^; ^1^H-NMR (400 MHz, DMSO-*d_6_*) δ 0.75 (t, *J* = 7.0 Hz, 3H), 1.35 (s, 9H), 2.39 (s, 3H), 3.32 (s, 6H), 3.68 (s, 3H), 3.71 (s, 3H), 3.88 (q, *J* = 7.0 Hz, 2H), 6.65 (d, *J* = 8.8 Hz, 1H), 6.78–6.83 (m, 2H), 7.14 (s, 1H), 7.22 (d, *J* = 8.8 Hz, 1H), 7.31 (d, *J* = 8.8 Hz, 1H), 7.47 (s, 1H), 7.60 (s, 1H), 10.15 (s, 1H); ^13^C-NMR (100 MHz, DMSO-*d_6_*) δ 10.4 (q), 13.5 (q), 27.8 (q), 32.6 (q), 32.9 (q), 54.5 (q), 55.1 (q), 58.9 (t), 80.7 (s), 101.2 (d), 102.3 (s), 102.8 (d), 108.9 (s), 110.4 (d), 111.2 (d), 111.7 (d), 112.4 (d), 117.5 (s), 122.7 (s), 126.0 (s), 126.7 (s), 128.0 (s), 130.4 (d), 131.2 (s), 132.2 (s), 135.7 (s), 137.5 (d), 153.6 (s), 154.6 (s), 155.4 (s), 163.8 (s), 186.5 (s). MS: *m/z* (%) = 614 [M^+^] (40), 558 (84), 514 (100), 499 (20), 452 (31), 188 (52). Anal. Calcd for C_34_H_38_N_4_O_7_: C, 66.43; H, 6.23; N, 9.11. Found: C, 66.79; H, 5.97; N, 9.23.

*Ethyl 1-[(tert-butoxycarbonyl)amino]-2-methyl-5-(5-chloro-1-methyl-1H-indol-3-yl)-4-[(5-chloro-1-methyl-1H-indol-3-yl)carbonyl]-1H-pyrrole-3-carboxylate* (**10d**). White solid; yield: 90%; mp: 195–199 °C; IR (nujol) ν 3257, 1754, 1678, 1637 cm^−1^; ^1^H-NMR (400 MHz, DMSO-*d_6_*) δ 0.76 (t, *J* = 7.2 Hz, 3H), 1.33 (s, 9H), 2.38 (s, 3H), 3.70 (s, 6H), 3.89 (q, *J* = 7.2 Hz, 2H), 7.08 (dd, *J* = 8.4 Hz, *J* = 1.8 Hz, 1H), 7.21 (dd, *J* = 8.4 Hz, *J* = 1.8 Hz, 1H), 7.29 (s, 1H), 7.38 (d, J = 8.4 Hz, 1H), 7.41–7.48 (m, 2H), 7.64 (s, 1H), 8.00 (d, J = 1.8 Hz, 1H), 10.26 (s, 1H); ^13^C-NMR (100 MHz, DMSO-*d_6_*) δ 10.3 (q), 13.5 (q), 27.7 (q), 32.7 (q), 33.0 (q), 59.0 (t), 80.8 (s), 102.2 (s), 108.8 (s), 111.4 (d), 112.2 (d), 117.0 (s), 119.0 (d), 120.3 (d), 121.3 (d), 122.6 (d), 122.8 (s), 124.3 (s), 125.4 (s), 126.6 (s), 126.8 (s), 128.6 (s), 132.4 (d), 134.6 (s), 135.7 (s), 136.2 (s), 138.9 (d), 154.5 (s), 163.6 (s), 186.3 (s). MS: *m/z* (%) = 622 [M^+^] (13), 566 (36), 522 (100), 507 (27), 357 (30), 328 (29), 192 (78). Anal. Calcd for C_32_H_32_Cl_2_N_4_O_5_: C, 61.64; H, 5.17; N, 8.99. Found: C, 61.77; H, 5.29; N, 8.91.

*Ethyl 1-[(tert-butoxycarbonyl)amino]-2-methyl-5-(5-bromo-1-methyl-1H-indol-3-yl)-4-[(5-bromo-1-methyl-1H-indol-3-yl)carbonyl]-1H-pyrrole-3-carboxylate* (**10e**). White solid; yield: 82%; mp: 201–205 °C; IR (nujol) ν 3261, 1755, 1693, 1637 cm^−1^; ^1^H-NMR (400 MHz, DMSO-*d_6_*) δ 0.77 (t, *J* = 7.0 Hz, 3H), 1.33 (s, 9H), 2.38 (s, 3H), 3.70 (s, 6H), 3.89 (q, *J* = 7.0 Hz, 2H), 7.19 (d, *J* = 8.4 Hz, 1H), 7.27 (s, 1H), 7.31–7.36 (m, 2H), 7.41 (d, J = 8.4 Hz, 1H), 7.56 (s, 1H), 7.63 (s, 1H), 8.17 (s, 1H), 10.26 (s, 1H); ^13^C-NMR (100 MHz, DMSO-*d_6_*) δ 10.3 (q), 13.6 (q), 27.8 (q), 32.6 (q), 33.0 (q), 59.0 (t), 80.8 (s), 102.1 (s), 108.8 (s), 111.9 (s), 112.3 (s), 112.6 (d), 114.7 (d), 116.9 (s), 122.0 (d), 122.8 (s), 123.3 (d), 123.8 (d), 125.1 (d), 125.4 (s), 127.4 (s), 129.2 (s), 132.3 (d), 134.8 (s), 135.9 (s), 136.1 (s), 138.8 (d), 154.5 (s), 163.6 (s), 186.3 (s). MS: *m/z* (%) = 710 [M^+^] (26), 612 (100), 566 (20), 471 (23), 401 (25), 252 (60), 236 (35), 181 (35). Anal. Calcd for C_32_H_32_Br_2_N_4_O_5_: C, 53.95; H, 4.53; N, 7.86. Found: C, 53.76; H, 4.65; N, 8.01.

### 3.2. Biology

#### 3.2.1. Methodology of the *in Vitro* Cancer Screen

The human tumor cell lines of the cancer screening panel are grown in RPMI 1640 medium containing 5% fetal bovine serum and 2 mM L-glutamine. For a typical screening experiment, cells are inoculated into 96 well microtiter plates in 100 µL at plating densities ranging from 5,000 to 40,000 cells/well depending on the doubling time of individual cell lines. After cell inoculation, the microtiter plates are incubated at 37 °C, 5% CO_2_, 95% air and 100% relative humidity for 24 h prior to addition of experimental drugs. After 24 h, two plates of each cell line are fixed *in situ* with TCA, to represent a measurement of the cell population for each cell line at the time of drug addition (Tz). Experimental drugs are solubilized in dimethyl sulfoxide at 400-fold the desired final maximum test concentration and stored frozen prior to use. At the time of drug addition, an aliquot of frozen concentrate is thawed and diluted to twice the desired final maximum test concentration with complete medium containing 50 µg/mL gentamicin. Additional four, 10-fold or ½ log serial dilutions are made to provide a total of five drug concentrations plus control. Aliquots of 100 µL of these different drug dilutions are added to the appropriate microtiter wells already containing 100 µL of medium, resulting in the required final drug concentrations. Following drug addition, the plates are incubated for an additional 48 h at 37 °C, 5% CO_2_, 95% air, and 100% relative humidity. For adherent cells, the assay is terminated by the addition of cold TCA. Cells are fixed *in situ* by the gentle addition of 50 µL of cold 50% (w/v) TCA (final concentration, 10% TCA) and incubated for 60 min at 4 °C. The supernatant is discarded, and the plates are washed five times with tap water and air dried. Sulforhodamine B (SRB) solution (100 µL) at 0.4% (w/v) in 1% acetic acid is added to each well, and plates are incubated for 10 min at room temperature. After staining, unbound dye is removed by washing five times with 1% acetic acid and the plates are air dried. Bound stain is subsequently solubilized with 10 mM trizma base, and the absorbance is read on an automated plate reader at a wavelength of 515 nm. For suspension cells, the methodology is the same except that the assay is terminated by fixing settled cells at the bottom of the wells by gently adding 50 µL of 80% TCA (final concentration, 16% TCA). Using the seven absorbance measurements [time zero, (Tz), control growth, (C), and test growth in the presence of drug at the five concentration levels (Ti)], the percentage growth is calculated at each of the drug concentrations levels. Percentage growth inhibition is calculated as:

[(Ti-Tz)/(C-Tz)] × 100 for concentrations for which Ti>/=Tz

[(Ti-Tz)/Tz] × 100 for concentrations for which Ti<Tz.

For further information to see NCI website [30].

## 4. Conclusions

In conclusion, five ethyl 1-[(*tert*-butoxycarbonyl)amino]-2-methyl-5-(1-methyl-1*H*-indol-3-yl)-4-[(1-methyl-1*H*-indol-3-yl)carbonyl]-1*H*-pyrrole-3-carboxylates derivatives **10a**–**e**, analogues of the marine alkaloid topsentin, in which the imidazole moiety was replaced by a pyrrole ring, were obtained. Only one of these compounds (compound **10e**) showed moderate activity against two cell lines belonging to the NCI CNS and non-small cell lung cancer sub-panels.
